# Irrigation solutions in open fractures of the lower extremities: evaluation of isotonic saline and distilled water

**DOI:** 10.1051/sicotj/2016031

**Published:** 2017-01-30

**Authors:** Olukemi Temiloluwa Olufemi, Adeolu Ikechukwu Adeyeye

**Affiliations:** 1 Department of Orthopaedic Surgery and Trauma, Ondo State Trauma and Surgical Centre, Medical Village Laje Road Ondo State Nigeria West Africa

**Keywords:** Open fractures, Irrigation, Distilled water, Saline

## Abstract

*Introduction*: Open fractures are widely considered as orthopaedic emergencies requiring immediate intervention. The initial management of these injuries usually affects the ultimate outcome because open fractures may be associated with significant morbidity. Wound irrigation forms one of the pivotal principles in the treatment of open fractures. The choice of irrigation fluid has since been a source of debate. This study aimed to evaluate and compare the effects of isotonic saline and distilled water as irrigation solutions in the management of open fractures of the lower extremities. Wound infection and wound healing rates using both solutions were evaluated.

*Methods*: This was a prospective hospital-based study of 109 patients who presented to the Accident and Emergency department with open lower limb fractures. Approval was sought and obtained from the Ethics Committee of the Hospital. Patients were randomized into either the isotonic saline (NS) or the distilled water (DW) group using a simple ballot technique. Twelve patients were lost to follow-up, while 97 patients were available until conclusion of the study. There were 50 patients in the isotonic saline group and 47 patients in the distilled water group.

*Results*: Forty-one (42.3%) of the patients were in the young and economically productive strata of the population. There was a male preponderance with a 1.7:1 male-to-female ratio. The wound infection rate was 34% in the distilled water group and 44% in the isotonic saline group (*p* = 0.315). The mean time ± *SD* to wound healing was 2.7 ± 1.5 weeks in the distilled water group and 3.1 ± 1.8 weeks in the isotonic saline group (*p* = 0.389).

*Conclusions*: It was concluded from this study that the use of distilled water compares favourably with isotonic saline as an irrigation solution in open fractures of the lower extremities.

## Introduction

Open fractures are widely considered as orthopaedic emergencies requiring immediate intervention. Approximately 3–4% of all fractures are open [1, 2]. Open fractures to the lower extremities are commonly seen at the Accident and Emergency department of the National Orthopaedic Hospital, Igbobi-Lagos with an average annual incidence of 1.5%.

The initial management of open fractures often affects the ultimate outcome. The most important initial step in the surgical wound management of open fractures is regarded as copious fluid irrigation along with meticulous debridement of surrounding contaminated soft tissues [1, 3, 4]. Conclusions drawn from Gustilo and Anderson’s classic article emphasize emergency treatment including copious irrigation and debridement [5].

Wound irrigation to remove debris and lessen bacterial contamination is an essential component of open fracture care [6, 7]. When performed properly, wound irrigation can enhance wound healing by reducing infection and its attendant morbidities.

Isotonic saline is regarded as the most appropriate and preferred irrigant because it is a nontoxic solution that does not damage the healing tissue [7–10]. Although widely used, its cost may preclude judicious use where large amounts are required and the need for a more affordable alternative cannot be overemphasized.

Distilled water is routinely used in the hospital laboratory on a daily basis for titration of solutions and evaluation of blood samples. It is produced by condensing steam, is nonpyrogenic and without antimicrobial agents or buffers. It is often used in irrigation as a less expensive alternative to isotonic saline, especially in developing countries [7–10].

The choice of irrigation solution, optimal amount required and method of delivery remain controversial [8, 11]. Given that wound irrigation is more dependent on mechanics than the antibacterial or chemical properties of the irrigation solutions, copious amounts are recommended and therefore less expensive and readily available solutions may be employed in the irrigation of open fractures.

This study aimed to evaluate and compare the effects of isotonic saline and distilled water as irrigating solutions in open injuries of the lower extremities.

## Materials and methods

This was a prospective, randomized, interventional, hospital-based study. Patients of all ages presenting at the Accident and Emergency department of the National Orthopaedic Hospital, Igbobi-Lagos with open fractures of the lower extremities were recruited over a period of 12 months. Approval was obtained from the Research and Ethics Committee of the Hospital prior to commencement of the study.

All patients who presented with open fractures were treated as emergency, using the Advanced Trauma Life Support (ATLS) protocol. Patients of all ages with Gustilo-Anderson I–IIIa open fractures of the lower extremities presenting within 24 h who consented were included in the study. Patients with potentially life-threatening injuries that required emergency interventions were excluded from the study.

Patients who met the inclusion criteria were then recruited into the study. An informed consent to participate in the study was taken. Using a simple ballot technique, patients were grouped into groups A and B. Patients in group A had wound irrigation done with isotonic saline, while group B patients had irrigation done with distilled water. Broad spectrum parenteral antibiotics were administered and continued for 72 h (Cefuroxime and Metronidazole). This was subsequently followed by oral forms in the presence of signs and symptoms of infection. Adequate analgesics were administered to patients, and opioids were used for this study. The wound was covered with sterile dressings. The fractured limb was immobilized with external splints, and appropriate plain radiographs were obtained.

Patients were then prepared for emergency wound debridement and copious irrigation in the theatre under aseptic conditions using general or regional anaesthesia. The operation site was cleansed with antiseptic lotion and sterile drapes applied. Dead tissues, nonviable tissues and devitalized desiccated bone were removed, and the edges of the wound excised. The wound was graded according to the Gustilo and Anderson classification after the debridement by the operating surgeon. The wound was then irrigated with at least three litres of isotonic saline or distilled water, according to guidelines listed in Table 1 which was pasted in the operating theatre. The wound was irrigated using a 20 mL piston syringe.

**Table 1.** Irrigation fluid volume used for each Gustilo grade.

Gustilo type	Irrigation
I	3 L of Isotonic saline/distilled water
II	6 L of Isotonic saline/distilled water
IIIa	9 L of Isotonic saline/distilled water

The timing and technique of fracture stabilization was determined by the consultant. Wound closure, where possible, was done using Prolene sutures on a cutting needle. Thereafter, sterile dressings were applied. Post-operatively, antibiotics and analgesics were continued as prescribed above. The affected limb was elevated in bed, and the vital signs monitored regularly.

The wound was inspected after 24 h, as well as the 3rd and 5th post-operative days for signs and symptoms of infection. The Cutting and Harding criteria were used to define the presence of infection clinically. These criteria included abscess, cellulitis, wound discharge, discolouration, delayed healing, friable granulation tissue, unexpected pain and tenderness, pocketing at the base of the wound, epithelial bridging, abnormal smell and wound breakdown [21]. Wound healing was defined, following wound inspection, as the presence of epithelial tissue covering the wound. These criteria had earlier been used by Griffiths et al. [24].

Wound swabs for microscopy, culture and sensitivity were taken to examine if signs of infection were present; otherwise sterile saline dressings were applied. Repeat wound debridement was done in the theatre as required. Continued use of antibiotics was determined by the sensitivity patterns of cultured organisms.

Wounds that showed evidence of infection by the 5th day were left open and appropriate dressings continued, until the wound was cleansed for a secondary wound closure. Sutures were removed between the 10th and 14th post-operative day, or if complete wound dehiscence occurred. Patients were followed up as either outpatients or inpatients.

The primary outcome measures of each patient group were wound infection and wound healing. The influence of time interval between injury and presentation, Gustilo grade, choice of irrigation solution and timing of wound closure on wound infection were evaluated.

Data was entered in a personal computer and analysed using the Statistical Package for Social Sciences (SPSS) version 17. Normally distributed numeric variables were summarized using their mean and standard deviations (Mean ± *SD*). For skewed numeric variables such as time intervals, the median and range were used. The chi-square (χ^2^) test was used to detect the association and comparison of proportions between categorical variables. The Fisher’s exact test was used in place of the chi-square test where expected frequencies were small or less than five. The relative risk (RR) of wound infection by irrigation fluid was calculated and the 95% Confidence Interval (CI) determined.

For independent samples, *t*-test was used to compare the mean of normally distributed numerical variables between the two study groups and where the variables were skewed, its nonparametric equivalent, the Mann-Whitney U test was used.

Finally, a multivariate binary logistic regression analysis was performed by entering wound infection as the dependent variable to determine its independent predictors. The generated odds ratio (OR) and 95% CI were approximated to the relative risk (RR). The results were presented in tables and appropriate charts. The level of significance was set at *p*-value < 0.05.

## Results

In total, 120 patients were recruited. Twenty-three patients were lost to follow-up and 97 patients were studied. They comprised 50 patients randomized into the Isotonic Saline (NS) group and 47 patients into the Distilled Water (DW) group.

**Figure 1.**
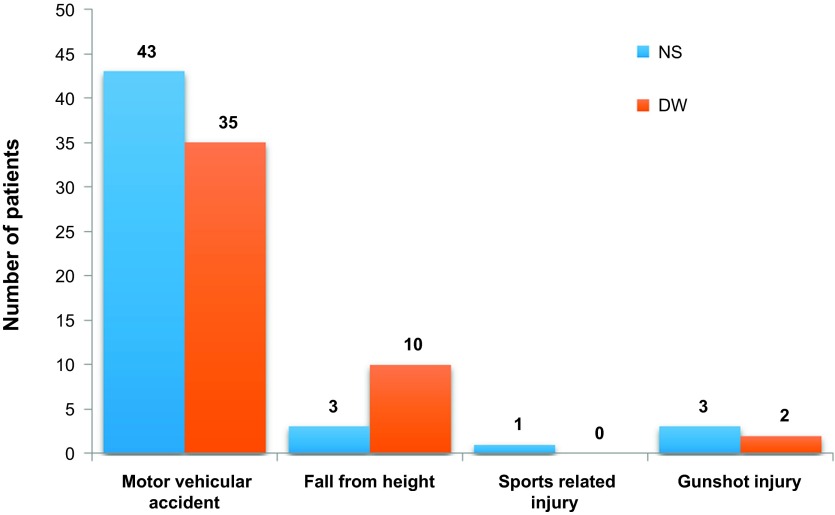
Mechanism of injury (*p* > 0.05).

**Figure 2.**
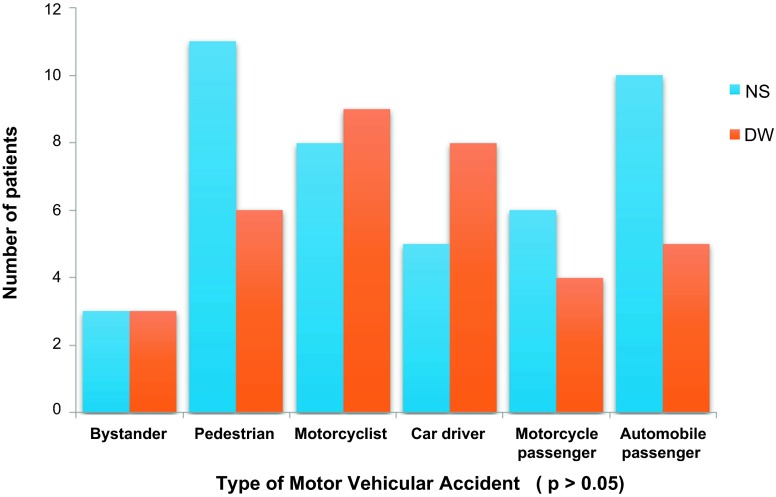
Type of motor vehicular accident.

The mean age of NS group was 37.1 ± 13.6 years. Although this was higher than the value for the DW group (34.6 ± 11.9 years), this difference was not significant (*p* = 0.330). The largest proportion of subjects in each of the two groups (32.0% NS and 53.0% DW group) belonged to the 30–39 year age group. There were significantly higher proportions of men in both study groups (*p* = 0.022). See Table 2. The tibia was affected in 44 out of the 94 patients studied. This accounts for the most common lower limb bone affected. The mechanism of injury of patients in both groups was compared and no significant difference was found (Figures 1 and 2).

**Table 2.** Demographic characteristics of the study population.

Variables	NS	DW	*t*	*df*	*p*-value
	Mean ± *SD*	Mean ± *SD*			
Age in years	37.1 ± 13.6	34.6 ± 11.9	0.979	95	0.330
Male	30.7 ± 11.0	32.7 ± 9.2	−0.788	59	0.434
Female	44.2 ± 12.9	40.2 ± 16.8	0.793	34	0.433
Age group (yrs)	*n* (%)	*n* (%)			
<20	2 (4.0)	4 (8.5)	7.073	4	0.132*
20–29	14 (28.0)	10 (21.3)			
30–39	16 (32.0)	25 (53.2)			
40–49	7 (14.0)	3 (6.4)			
50+	11 (22.0)	5 (10.6)			
Gender					
Male	26 (52.0)	35 (74.5)	5.240	1	0.022*
Female	24 (48.0)	12 (25.5)			
Affected bones					
Femur	20 (40)	10 (21.3)			
Tibia	18 (36)	26 (55.3)			0.145
Ankle	12 (24)	11 (23.4)			

NS – Isotonic saline group, DW – Distilled water group; *t* – Independent samples *t*-test, *df* – degrees of freedom, *Chi-square test applied.

Among patients who developed wound infection, there was no significant difference in both groups (RR 0.774, 95% CI 0.466–1.283). Of the 22 NS patients with wound infection, 9 (40.9%) developed osteomyelitis compared to five (31.2%) out of the 16 with wound infection among the DW group. This difference was not statistically significant (χ^2^ = 0.072, *df* = 1, *p* = 0.788).

*Staphylococcus* spp. was the commonest organism in six out of 16 (37.5%) of the NS group with a positive culture growth. It was also the commonest in three out of seven (42.9%) in the DW group. *Klebsiella* spp*.* and *Proteus* spp. were the commonest gram-negative organisms cultured.

Primary wound closure was done in 31 (62.0%) NS patients and 33 (70.2%) DW patients. Other methods of wound closure used were delayed primary closure and split thickness skin graft. Wound healing rates in both groups were not significantly different though the mean time to wound healing was 3.1 ± 1.8 weeks in the NS group and 2.7 ± 1.5 weeks in the DW group. See Table 3.

**Table 3.** Assessment of wound among study subjects.

Variables	NS	DW	χ^2^	*df*	*p*-value
	*n* (%)	*n* (%)			
Wound closure					
Primary closure	31 (62.0)	33 (70.2)	1.411	2	0.494
Delayed primary closure	14 (28.0)	12 (25.5)			
Split thickness skin graft	5 (10.0)	2 (4.3)			
Wound infection					
Absent	28 (56.0)	31 (66.0)			
Present	22 (44.0)	16 (34.0)	1.008	1	0.315
	RR 0.774, 95% CI 0.466–1.283				
Wound healing					
Two weeks	29 (58.0)	30 (63.8)	0.960	2	0.619
Four weeks	15 (30.0)	14 (29.8)			
Eight weeks	6 (12.0)	3 (6.4)			
Time to wound healing (weeks)					
Mean ± *SD*	3.1 ± 1.8	2.7 ± 1.5	−0.862		0.389*
Median (range)	2.0 (2.0–8.0)	2.0 (2.0–8.0)			

* Mann-Whitney U test applied.

A multivariate binary logistic regression analysis showed that only the grade of fracture (Gustilo Grade II) was found to be independently associated with wound infection. Patients with Gustilo type II fracture were significantly less likely to have wound infection compared to those with Grade IIIa (RR 0.078, 95% CI 0.023–0.262). Although the DW group had a 10% reduced risk of wound infection compared to the NS group, this was not statistically significant (RR 0.9, 95% CI 0.279–2.906). See Table 4.

**Table 4.** Multivariate logistic regression for predictors of wound infection.

Variables	RR (95% CI)	*p*-value
Age group (yrs)		
<20	0.259 (0.009–7.560)	0.432
20–29	0.752 (0.126–4.501)	0.755
30–39	0.512 (0.095–2.767)	0.437
40–49	0.585 (0.064–5.326)	0.634
50+	1	
Irrigation fluid		
Distilled water	0.9 (0.279–2.906)	0.860
Isotonic saline	1	
Gustilo grade		
Grade I	0.000	0.999
Grade II	0.078 (0.023–0.262)	0.000
Grade IIIa	1	
Time interval between injury and presentation (hrs)		
<1	0.000	0.999
2–6	0.275 (0.073–1.039)	0.057
7–12	0.456 (0.107–1.948)	0.289
13–24	1	
Time interval between presentation and initial debridement (hrs)		
3–5	0.000	0.999
6–12	4.302 (0.244–75.878)	0.319
13–24	2.518 (0.161–39.306)	0.510
>24	1	

## Discussion

The fate of an open fracture depends a lot on the initial treatment administered to the wound [12]. In the treatment of open fractures, copious use of solution in order to reduce the degree of pollution by contaminants is a critical step in the debridement of the wound [6]. The choice of irrigation solution is therefore very important in the final outcome of a wound.

The age of patients in this study ranged from 18 to 73 years, however, male patients in the active age group of 30–39 years were predominantly affected. This finding is similar to the work done in Nigeria by Ikem et al. [13] and Thanni and Kehinde [14]. They found that males within this age group formed a larger portion of patients with open fractures. Injuries, especially to the active working population in any country, bear a significant socioeconomic burden on the community.

Most patients in this study had open fractures from traffic accidents. Others were secondary to gunshots, falls from height and sport injuries. Road accidents are leading causes of trauma injuries in the world and one of the leading causes of fractures in Nigeria [15]. Most of these injuries occur in unsterile environments and lead to contamination of the wound from the environment, thus making wound irrigation essential to open fracture care.

The most frequently injured lower limb bone in this study was the tibia, accounting for a total of 44 injuries (45.7%), while femoral and ankle fractures followed at 23.4% and 25.5%, respectively. The subcutaneous nature of the tibia in a bipedal man as compared to the large protective tissue envelope of the femur may account for this, as documented in the literature [16–18].

We noted that the Gustilo grade was significantly associated with the rate of wound infection in both groups. It was noted that higher Gustilo grades were associated with higher wound infection rates (*p* = 0.000 in the NS group and *p* = 0.025 in the DW group). Gustilo and Anderson, Patzakis and Wilkins and other researchers documented a similar finding in their studies [5, 19, 20]. This may be as a result of the extent of tissue damage and tissue necrosis. The total extent of tissue injury may not be apparent at the time of first surgery leading to retention of necrotic tissue which is a nidus of infection. Wound irrigation on its own would serve to reduce contamination while debridement would remove dead tissue.

Wound infection was assessed using the Cutting and Harding criteria [21]. When the infection rates were compared, wound infection was more common among patients who used isotonic solution. Despite this finding, there was no significant difference in the infection rates in patients who had isotonic saline for wound irrigation and those who had distilled water for wound irrigation (*p* = 0.315). This suggests that irrigation fluid volume, rather than the type of irrigation fluid, is the more important factor in the reduction of bacteria load in open fractures. Similar findings have been noted in earlier studies [7, 10, 22, 23].

Wound healing was defined as epithelial covering of the wound as described earlier in a study by Griffiths et al. [24]. The mean time to wound healing was 3.1 weeks in the isotonic saline group and 2.7 weeks in the distilled water group. Although the wound healing rate appeared faster in the distilled water group, this finding was not statistically significant (*p* = 0.389). Isotonic saline is favoured as it does not interfere with the normal healing process. However, findings in this study are similar to the findings of Museru et al. [7], where they found that distilled water did not negatively affect wound healing. We postulate that this can be alluded to the fact the hypotonic effect of distilled water is transient.

Guiding principles in open fractures are constantly evolving, however, it is widely accepted that wound irrigation plays a very fundamental role. The irrigating solution acts simply as a mechanical cleanser, therefore the volume used is crucial. Isotonic saline is generally preferred because, due to its tonicity, it is less toxic to tissues than other commercial irrigants. It may be argued that the hypotonicity of distilled water may have the potential for cellular damage and possibly delayed wound healing or impaired cellular function which could ultimately result in increased infection rates [7, 10, 24]. This study demonstrated that distilled water is as effective as isotonic saline in irrigating open fractures of the lower extremities.

## Conclusion and recommendations

Distilled water and isotonic saline are both effective irrigation solutions. There is no significant difference in wound infection rates or wound healing rates using either distilled water or isotonic saline for wound irrigation in open fractures of the lower extremities.

We concluded from this study that distilled water should be used as an alternative to isotonic saline for wound irrigation in open fractures of the lower extremities.

## Conflict of interest

OOT and AAI certify that they have no financial conflict of interest (e.g., consultancies, stock ownership, equity interest, patent/licensing arrangements, etc.) in connection with this article.
